# Editorial Notes

**Published:** 1930

**Authors:** 


### Editorial Notes

**British Association Meeting**

For the fourth time in its history the British Association for the Advancement of Science has met in Bristol.

The previous occasions were in 1836, 1875 and 1898. On the last occasion Dr. Bertram Rogers acted as one of the local Secretaries, and he achieved the distinction (together with the burden) of occupying the same office in 1930. The success of the meeting must have been most gratifying to all the officers of the Association both of the General Council and the Local Committees. The membership reached the high figure of 2,650, and the satisfaction expressed with all the arrangements was universal. The University buildings provided wonderful accommodation for the comfort of members and for the meetings of the various sections. Professor F. O. Bower, F.R.S., opened the Session with an inaugural address on "Size and Form in Plants" to a crowded and appreciative audience in the Colston Hall on Wednesday, 3rd September, whilst the final ceremony of the meeting was a special congregation of the University to confer upon him, as President of the Association, the honorary degree of D.Sc. Apart from the papers read at the various sections, there were many public lectures of the highest importance. The most interesting to members of the Medico-Chirurgical Society was undoubtedly that delivered by Sir Arthur Keith on "John Beddoe." This great anthropologist, to whose labours Sir Arthur Keith paid just tribute, was one of the first members of this Society and a man whose pre-eminence was amply recognized in Bristol during his lifetime. Sir Arthur made the suggestion that a Chair of Anthropology should be founded in memory of Beddoe. It is hoped that the University will carry the suggestion into effect. Anthropology is far more than a mere delving into antiquity, it is one of the most living of the "humanities," and promises to furnish information of the utmost value to Sociology.

The numerous excursions into the country surrounding Bristol aroused the most enthusiastic praise from the members of the Association. The surprise expressed by some recalled an entry in the Diary of Joseph Farington (dated 1808, February 28th): "Fuseli was last week at Clifton near Bristol and raved about the romantic scenery, saying 'It was the finest thing in the kingdom, sublime, etc.' West repeated what he had often said before, 'That the country included within twenty miles of Bath contained more variety of landscape and noble scenes than any other county that could be mentioned.'" Judging from the comments of our visitors, the verdicts of Fuseli and Benjamin West still hold good.

**The late Rev. H. N. Burden, Warden of the National Institutions for Persons requiring Care and Control.**

INSPIRED partly, no doubt, by the very nature of his profession, but infinitely more by a personal sense of humanitarianism, the late Reverend H. N. Burden early in life embarked upon a career of practical philanthropy. Accident of opportunity, combined with friendships with those similarly interested, first directed Mr. Burden's attention to the pitiful lot of the confirmed inebriate. To think was to act, and accordingly a small Home for Inebriates was opened at Horfield, Bristol, within the shadow of the prison gates, and this was soon followed by the acquisition, through his efforts and for the same purpose, of what is now the Brentry Colony for Mental Defectives.

From this small beginning have sprung the magnificent—and the adjective is not ill-chosen—Homes for the Mentally Defective now to be found within and around the City of Bristol. Mr. Burden found, as others have since done, that inebriety is more frequently the result of mental deficiency than its cause, and so the transition from one to the other was a natural and almost inevitable result of Mr. Burden's broad-minded philanthropy for the benefit of his less fortunately endowed fellow-beings.

From the time Mr. Burden first joined the Royal Commission on Mental Deficiency more than twenty years ago he unceasingly devoted his time, his money, and his undoubted business acumen to the betterment of the study, housing, and treatment of the mentally defective child and adolescent. Family mansions within the Bristol area and other parts of the country were, as they came into the market, gradually acquired by him for the benefit of the nation's unfortunates. The splendid properties of Stoke Park, Leigh Court, Stapleton Park (known as Beech House), Whittington Hall, and others of less note, were purchased privately by Mr. Burden without any assistance from rates, donations, or public or private grants of any sort, and devoted to his great objective, and all without ostentation, publicity, or proclamation from the house-tops of the grandeur of the conception and the nobility of the charity. So quietly indeed did Mr. Burden achieve his good deeds that his intentions are not yet quite clearly recognized even in Bristol. But these were at least two-fold; one, the foundation of a series of national institutions for national purposes, and the other, the prosecution of study and research into mental deficiency. His first main objective is already largely achieved, for the several properties have been vested in trustees under a trust deed with instructions for their use for any national philanthropic purpose.

The cause of the mentally defective Mr. Burden had ever at heart and his second objective, achieved almost on his death-bed, was the organization on a liberal scale of medical and scientific research into mental deficiency, to be associated, with the consent of the authorities, with the University of Bristol. The names of the men to whom this task of research into a great and pressing national problem have already been announced, to which now falls to be added that of Dr. R. G. Gordon of Bath. To them there is now given a great opportunity, and no one, knowing to whom this task has been entrusted, can doubt that their ultimate researches into the origins, causes, concomitants, and possible remedial measures of mental deficiency will do credit to the memory of the late Warden of Stoke Park Colony, and will worthily uphold the traditions and prestige of the City and University of Bristol.

As we go to press we learn that the Trustees have, with the approval of the Secretary of State, appointed Mrs. Rosa Gladys Burden, of Clevedon Hall, to be Warden of the National Institutions in succession to the late Rev. Harold Nelson Burden.

R. J. A. B.

